# Regulation of Proinflammatory Mediators via NF-**κ**B and p38 MAPK-Dependent Mechanisms in RAW 264.7 Macrophages by Polyphenol Components Isolated from Korea *Lonicera japonica THUNB*

**DOI:** 10.1155/2012/828521

**Published:** 2012-05-07

**Authors:** Kwang-Il Park, Sang-Rim Kang, Hyeon-Soo Park, Do Hoon Lee, Arulkumar Nagappan, Jin A Kim, Sung Chul Shin, Eun Hee Kim, Won Sup Lee, Hyon-Jong Chung, Su Jin An, Gon Sup Kim

**Affiliations:** ^1^Research Institute of Life Science and College of Veterinary Medicine, Gyeongsang National University, Gazwa, Jinju 660-701, Republic of Korea; ^2^Department of Biological Engineering, School of Natural Science, Kyonggi University, Yeongtong, Suwon 443-760, Republic of Korea; ^3^Korea National Animal Research Resource Center and Korea National Animal Bio-Resource Bank, Gyeongsang National University, Gazwa, Jinju 660-701, Republic of Korea; ^4^Department of Chemistry and Research Institute of Life Science, Gyeongsang National University, Jinju 660-701, Republic of Korea; ^5^Department of Nursing Science, International University of Korea, Jinju 660-759, Republic of Korea; ^6^Department of Internal Medicine, Institute of Health Sciences, Gyeongsang National University School of Medicine, Jinju 660-702, Republic of Korea; ^7^Gyeongnam Regional Cancer Center, Gyeongsang National University Hospital, Jinju 660-702, Republic of Korea

## Abstract

*Lonicera japonica THUNB.*, which abundantly contains polyphenols, has been used as a traditional medicine for thousands of years in East Asian countries because of the anti-inflammation properties. This study aimed to investigate the anti-inflammatory mechanism of polyphenol components isolated from Korea *L. japonica T.* by nuclear factor-kappaB (NF-**κ**B) and mitogen-activated protein kinases (MAPKs) pathway. Polyphenols significantly decreased lipopolysaccharide- (LPS-) induced mRNA and protein expression of inducible nitric oxide synthase and cyclooxygenase-2, as well as mRNA expression of tumor necrosis factor-alpha, interleukin- (IL-) 1**β**, and IL-6. Moreover, polyphenols inhibited nuclear translocation of NF-**κ**B p65, phosphorylation/degradation of the inhibitor of **κ**B, and phosphorylation of p38 MAPK, whereas the extracellular signal-regulated kinase and Janus N-terminal kinase were not affected. These results indicate that polyphenol components isolated from Korea *L. japonica T.* should have anti-inflammatory effect on LPS-stimulated RAW 264.7 cells through the decrease of proinflammatory mediators expression by suppressing NF-**κ**B and p38 MAPK activity.

## 1. Introduction

*Lonicera japonica THUNB.* (Caprifoliaceae) has been used in East Asian countries including Korea, China, and Japan as the treatment of inflammation and tumor for thousands of years. Crude extracts of *L. japonica* have hepatoprotective and anti-inflammatory effects associated with suppression of nuclear factor-kappaB (NF-*κ*B) activation through reduction of I-kappaB degradation in lipopolysaccharide- (LPS-) challenged rats [1]. *L. japonica T.* extract exhibits neuroprotective effects associated with the suppression of hydrogen peroxide-induced apoptosis via phosphorylation of mitogen-activated protein kinases (MAPKs) and phosphoinositide 3-kinase (PI3K)/Akt in neuroblastoma cells [2]. A number of compounds isolated from *Lonicera* species, including luteolin, quercetin, biflavonoids, and dicaffeoylquinic acid, have various pharmacological properties such as antimicrobial, antioxidative, antiviral, and anti-inflammatory effects [3, 4].

Inflammation encompasses multiple processes by an organism in response to injury that is related to harmful stimuli such as infection by a pathogen, exposure to endotoxin (e.g., LPS), or chemical exposure. Inflammatory responses are pivotal in host defense against stimuli by activating immune cells, in an effort to maintain homeostasis [5]. Macrophages play a critical role in the initiation of inflammatory and immune responses by releasing proinflammatory mediators such as tumor necrosis factor-alpha (TNF-*α*), interleukin-6 (IL-6), cyclooxygenase-2 (COX-2), and inducible nitric oxide synthase (iNOS) [6]. When macrophages are activated by stimuli, Toll-like receptor 4 (TLR4) involvement with the myeloid differentiation factor (MyD88) preludes the MyD88-dependent pathway triggering of the activation of transforming-growth-factor-beta- (TGF-*β*-) activated kinase 1 (TAK1). TAK1 activation causes the phosphorylation of MAPKs and I*κ*B kinase (IKK). Eventually, NF-*κ*B is activated by these cell signals [7]. However, chronic inflammation leads to the upregulation of pro-inflammatory mediators in affected cells and overexpression of the proinflammatory mediators such as iNOS and COX-2, and various cytokines including TNF-*α*, IL-1*β*, and IL-6 can cause many inflammation-related diseases, including atherosclerosis, colitis, rheumatoid arthritis, inflammatory bowel disease, and cancer [8–10].

NF-*κ*B plays a crucial role in chronic inflammatory diseases [11–13]. NF-*κ*B is a heterodimer composed of p50 and p65, which functions principally as a transcriptional activator. NF-*κ*B is changed to the active form by cytokines, LPS, and oxidative stress, whereupon it regulates COX-2 and iNOS through the initiation of transcription of target genes. However, the inherent mechanism underlying the effect of *L. japonica T. *on LPS-induced inflammation remains incompletely understood.

In the present study, we investigated whether polyphenol components isolated from *L. japonica* T. have anti-inflammatory activity via the production of COX-2, iNOS, and cytokines such as IL-1*β*, TNF-*α*, and IL-6 by inactivation of the NF-*κ*B and MAPK pathways in LPS-treated RAW 264.7 macrophages.

## 2. Materials and Methods

### 2.1. Extraction and Isolation of Polyphenol Components of *Lonicera japonica T.*

*L. japonica T. *was obtained from the Animal Bio-Resources Bank. The lyophilized plant material (100 g) was ground into powder and extracted in 500 mL of 70% methanol at 50°C for 12 h. After the extract was filtered in a Büchner funnel under reduced pressure and concentrated to 100 mL under reduced pressure at condition of 40°C using a rotator evaporator, the concentrated mixtures were reconstituted in methanol (0.01 g/mL) and stored at −20°C until analysis. The concentrated extracts were washed with *n*-hexane (100 mL), extracted with ethyl acetate (100 mL). After three extraction cycles, the organic layer was dried over anhydrous MgSO_4_. The solvent was removed under reduced pressure. The sticky residue was loaded onto a silica gel column (3.7 × 20 cm) and eluted with methanol/dichloromethane (1 : 5, 300 mL). The solvent was abolished to give different mixtures of polyphenols according to the original plant components (0.64% of the dried leaves; 0.28% of stems; 0.35% of flowers). The mixtures of polyphenols were reconstituted in methanol (0.01 g/mL), filtered through 0.45 *μ*m cellulose membranes, transferred into siliconized vials, and stored at −20°C until HPLC analysis.

High-performance liquid chromatography (HPLC) was conducted using a 1100 series LC system equipped with a G1322A degasser, G1312A pump, G1313A autosampler, and a G1316A oven (Agilent Technologies, Palo Alto, CA, USA). Chromatographic separation was performed on a Zorbax StableBond Analytical SB-C_18_ column (4.6 × 250 mm, 5 *μ*m; Agilent Technologies). The binary solvent system consisted of 1% aqueous formic acid (A) and methanol (B) with a linear gradient of 10–50% B over 50 min, decreased to 10% B over 10 min, followed by 10 min of isocratic elution at 10% B. The flow rate was 0.5 mL/min, with the column temperature maintained at 30°C and an injection volume of 10 mL in each experiment. PAD spectra were measured over the wavelength range of 240–600 nm in a 2 min step. Chromatographic data were collected and manipulated using ChemStation, Rev.B.0302. Tandem mass spectrometry (MS/MS) experiments were conducted on a 3200 Q TRAP LC-MS/MS system (Applied Biosystems, Foster City, CA, USA) with a Turbo V source and a Turbo Ion Spray probe operation at 450°C. The mass spectrometer was operated in the negative ion mode. BioAnalyst, version 1.4.2, and Analyst software, version 1.4.2 (AB Sciex, Zagreb, Croatia) were used for instrumental control and data acquisition, respectively. Nitrogen at a pressure of 60 psi was used as a nebulizing and drying gas. The capillary voltage was set at 5.5 kV with a source temperature of 400°C. The mass spectra were obtained over m/z 100–1,500 with a step size of 0.1 amu.

### 2.2. Chemicals

Fetal bovine serum (FBS), antibiotics (streptomycin/penicillin), and Dulbecco's modified Eagle's medium (DMEM) were purchased from Gibco (Grand Island, NY, USA) and Hyclone (Logan, UT, USA). *Escherichia coli* O111:B4 LPS and (3-(4,5-dimethyl-2yl)-2,5-diphenyltetrazolium bromide) (MTT) were purchased from Sigma-Aldrich (St. Louis, MO, USA). Antibodies to anti-COX-2 and iNOS were purchased from Santa Cruz Biotechnology (Santa Cruz, CA, USA), and anti-*β*-actin was purchased from Chemicon (Temecula, CA, USA). An antibody sampler kit of phospho-ERK1/2, p38, c-Jun N-terminal kinase (JNK) MAPKs, phospho-I*κ*B, I*κ*B, and NF-*κ*B p65 were obtained from Cell Signaling Technology (Danvers, MA, USA).

### 2.3. Macrophage Cell Culture and Treatment

RAW 264.7 murine macrophage cells (Korea Cell Line Bank, Seoul, Republic of Korea) were cultured in DMEM (2 mM L-glutamine, 100 units/mL penicillin and 100 *μ*g/*μ*L, and 10% fetal bovine serum) in a 5% CO_2_ humidified incubator at 37°C. RAW 264.7 cells were grown in six-well plates at a density of approximately 1 × 10^6^ cells per well. The polyphenol compounds were dissolved in dimethylsulfoxide (DMSO) and filtered through 0.45 *μ*m cellulose membranes. Cells were pretreated with the polyphenol components isolated from *L. japonica T.* at various concentrations (10, 50, 100, and 200 *μ*g/mL) and then stimulated with 1 *μ*g/mL LPS for 30 min, 6 h, and 24 h.

### 2.4. Assay for Cell Viability

The cell viability inhibitory effect of polyphenols on RAW 264.7 cells was assessed by the mitochondrial respiration-dependent reduction method of MTT to formazan. Cells seeded in 12-well plates (1 × 10^4^ cells/well) were pretreated with various concentrations of polyphenols (10, 50, 100, 200, and 300 *μ*g/mL) and then treated with LPS (1 *μ*g/mL) at 37°C in 5% CO_2_ for 24 h. After treatment, 100 *μ*L of MTT (5 mg/mL) dissolved in DMEM was added to each well, followed by incubation for 3 h. The medium was aspirated, and the formazan crystals were dissolved in 500 *μ*L of DMSO for 15 min. The optical density of each well was measured at 540 nm. The assay was carried out in triplicate.

### 2.5. Western Blot Analysis

Raw 264.7 cells were seeded in six-well plates at a density of approximately 1 × 10^6^ cells per well and incubated with or without LPS in the presence or absence of polyphenols. The cells were washed twice with ice-cold PBS and resuspended in lysis buffer containing 50 mM Tris-HCl (pH 8.0), 150 mM NaCl, 0.5% sodium deoxycholate, 0.1% sodium dodecyl sulfate (SDS), 1% NP-40, protease inhibitor cocktail, 0.5 M EDTA, and phosphatase inhibitor. The cell lysate was obtained from the supernatant after centrifugation at 13,000 rpm, 4°C, and 30 min. The protein concentration was determined using a Bradford assay kit (Bio-Rad, Hercules, CA, USA). Equal amounts of proteins were blotted onto an Immobilon-P 0.45 mm polyvinylidene fluoride (PVDF) membrane (Millipore, Billerica, MA, USA) following separation by 10% SDS-polyacrylamide gel electrophoresis (SDS-PAGE). The transferred proteins were incubated overnight at 4°C with a dilution of primary antibody (anti-iNOS, COX-2, phospho-I-*κ*B, NF-*κ*B p65, phospho-ERK1/2, phospho-JNK, or phospho-p38) and *β*-actin and then washed five times with Tris-buffered saline containing 0.5% Tween-20 (TBS-T, pH 7.4) for 10 min. After washing, the blots were incubated with horseradish peroxidase-conjugated secondary antibody for 1 h and again washed five times in TBS-T. The membranes were developed using an ECK kit (GE Healthcare Life Sciences, Buckinghamshire, UK). The intensity of each band was quantitatively determined using Image J software (http://rsb.info.nih.gov).

### 2.6. RNA Extraction and Reverse Transcription-Polymerase Chain Reaction (RT-PCR)

RAW 264.7 cells were pretreated with polyphenols for 1 h and then stimulated with 1 *μ*g/*μ*L LPS for 6 h. Total RNA was isolated using TRIzol reagent (GeneALL Biotechnology, Seoul, Republic of Korea) and reverse-transcribed into cDNA by commercially available cDNA synthesis kits (iScript cDNA Synthesis Kit; Bio-Rad) according to the manufacturer protocol. cDNA (1 *μ*g/*μ*L) was used to perform RT-PCR. The primer sequences used for quantification of iNOS, COX-2, TNF-*α*, IL-1*β*, IL-6, and glyceraldehyde 3-phosphate dehydrogenase (GAPDH) and the PCR conditions were the same as previously described [14]. The PCR products were separated by 1.5% agarose gel electrophoresis and visualized by ethidium bromide (EtBr) staining. The gels were then analyzed with ultraviolet transillumination. The quantity of each mRNA was calculated by using Image J software and normalized to the amount of the housekeeping GAPDH gene.

### 2.7. Statistical Analysis

All experiments were reiterated at least three times. The results of multiple observations are expressed as the mean ± SD. Statistical significance was determined by one-way analysis of variance (ANOVA) using SPSS version 10.0 for Windows (SPSS, Chicago, IL, USA) for multiple comparisons. A value of *P* < 0.05 was considered statistically significant.

## 3. Results

### 3.1. Characterization and Quantification of Polyphenol Components in *Lonicera japonica THUNB.*

**Figure 1:**
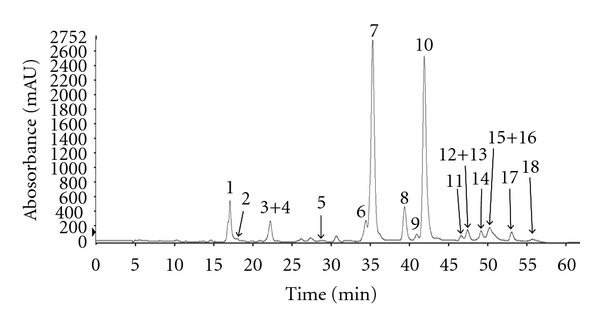
HPLC chromatogram and chemical structures of polyphenol components isolated from *L. japonica T.* HPLC chromatogram of* L. japonica T.*: (1) caffeoylquinic acid dimer, (2) caffeoylquinic acid, (3) caffeoylglycerol, (4) 5-p-coumaroylquinic acid, (5) feruloylquinic acid, (6) dicaffeoylquinic acid, (7) dicaffeoylquinic acid, (8) kaempferol 3-O-glucoside, (9) kaempferol-*O*-rutinoside, (10) dicaffeoylquinic acid, (11) apigenin-7-O-glucoside, (12) apigenin rutinoside, (13) feruloyl caffeoylquinic acid, (14) trihydroxymethoxyflavone, (15) kaempferol, (16) isorhamnetin glucoside, (17) caffeic acid derivative, and (18) feruloyl caffeoylquinic acid.

Polyphenol components were isolated from *L. japonica T. *using HPLC. Based on the HPLC retention time characteristics and the ultraviolet-visible spectra of standard compounds in a library, the components isolated from *L japonica T.* were present as 18 peaks and were identified (Figure 1). The 18 polyphenols that were comprised of 11 hydroxycinnamic acids and seven flavonoids were recorded at 280 nm. The quantification values of the 18 components are shown in Table 1. 

### 3.2. Effect of Polyphenols on Cell Viability

The cytotoxicity of polyphenols isolated from *L. japonica T.* on the viability of RAW 264.7 cells was measured in the concentration range of 10–300 *μ*g/mL using the MTT assay. Cell viability was not significantly changed by any of the polyphenol concentrations compared with the negative control (data not shown). Cotreatment of polyphenols and LPS (1 *μ*g/mL) was not cytotoxic at any of the concentrations used. We used 200 *μ*g/mL for maximum concentration of polyphenols isolated from *L. japonica T.*, and this concentration was used in subsequent experiments.

### 3.3. Inhibitory Effect of Polyphenols on COX-2 mRNA and Protein Expression in LPS-Stimulated RAW 264.7 Cells

**Figure 2:**
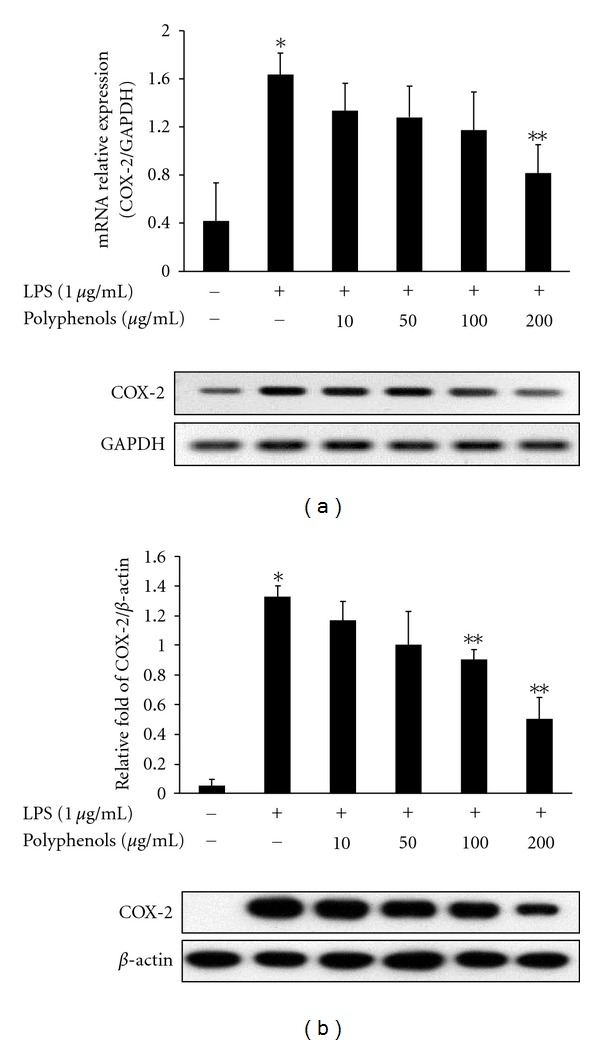
Effect of polyphenols on COX-2 mRNA and protein expression in RAW 264.7 macrophages. RAW 264.7 cells were pretreated with the indicated concentrations of polyphenols for 1 h and treated with LPS (1 *μ*g/mL). (a) After 6 h, total RNA of the cells was subjected to RT-PCR. (b) After 24 h, equal amounts of protein were subjected to 10% SDS-PAGE. Expression of COX-2 proteins was determined by Western blot analysis. Data are the mean ± SD of triplicates. The asterisk (*) indicates a significant difference from the control group (*P* < 0.05) and **indicates significant difference from the LPS-treated group (*P* < 0.05).

LPS is associated with a marked increase in COX-2 expression in RAW 264.7 cells. This was also the case presently; expression of COX-2 mRNA was markedly increased upon exposure to LPS for 6 h. Pretreatment with 200 *μ*g/mL polyphenols decreased the mRNA expression of LPS-induced COX-2 (Figure 2(a)). Also, studies were conducted to determine whether the expression of COX-2 protein paralleled its mRNA. RAW 264.7 cells treated with LPS displayed a markedly induced expression of COX-2 protein, while its protein was not detectable in unstimulated cells. In response to LPS treatment, the expression of COX-2 protein was significantly increased and polyphenols markedly suppressed COX-2 protein induction at concentrations of 100 and 200 *μ*g/mL (Figure 2(b)).

### 3.4. Inhibitory Effect of Polyphenols on iNOS mRNA and Protein Expression in LPS-Stimulated RAW 264.7 Cells

**Figure 3:**
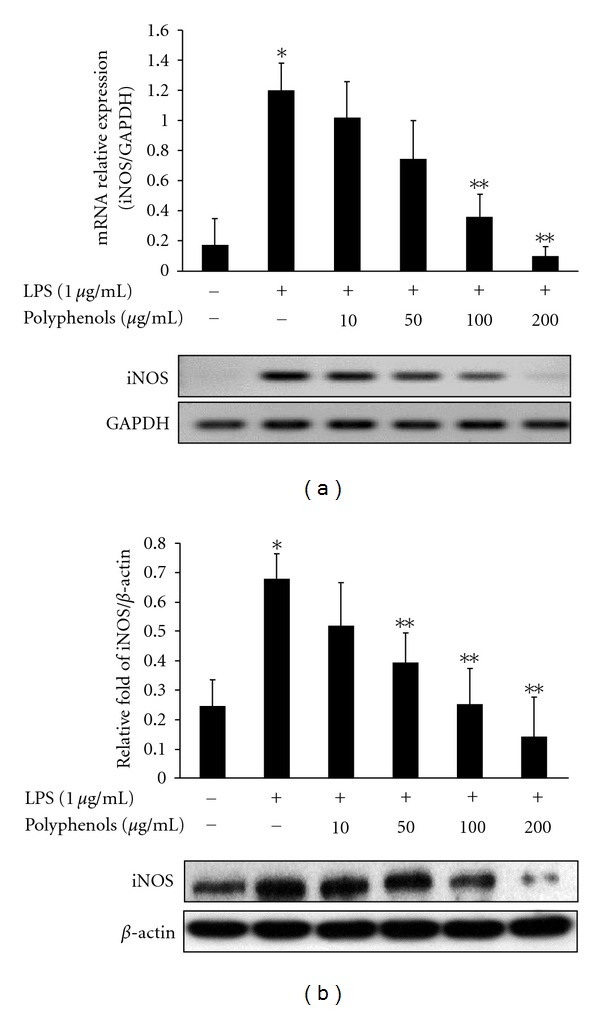
Effect of polyphenols on iNOS mRNA and protein expressions in RAW 264.7 macrophages. RAW 264.7 cells were pretreated with the various concentrations of polyphenols for 1 h and treated with LPS (1 *μ*g/mL). (a) After 6 h, total RNA of the cells was subjected to RT-PCR. (b) After 24 h, equal amounts of protein were subjected to 10% SDS-PAGE. Expression of COX-2 proteins was determined by Western blot analysis. Data are the mean ± SD of triplicates. The asterisk (*) indicates a significant difference from the control group (*P* < 0.05) and **indicates significant difference from the LPS-treated group (*P* < 0.05).

As shown in Figure 3(a), LPS induced a significant increase in expression of iNOS protein and mRNA. However, cotreatment of polyphenols and LPS inhibited the expression of iNOS mRNA at 100 and 200 *μ*g/mL of concentrations in LPS-stimulated RAW 264.7 cells. Furthermore, based on this result, we assessed whether polyphenols affected the expression of iNOS protein. RAW 264.7 cells induced high levels of iNOS protein when treated with LPS for 24 h, while the expression of iNOS protein was hardly detectable in un-stimulated cells. Addition of polyphenols significantly inhibited the LPS-induced iNOS protein production in a dose-dependent manner (Figure 3(b)).

### 3.5. Inhibitory Effect of Polyphenols on mRNA Expression of Proinflammatory Cytokines in LPS-Stimulated RAW 264.7 Cells

**Figure 4:**
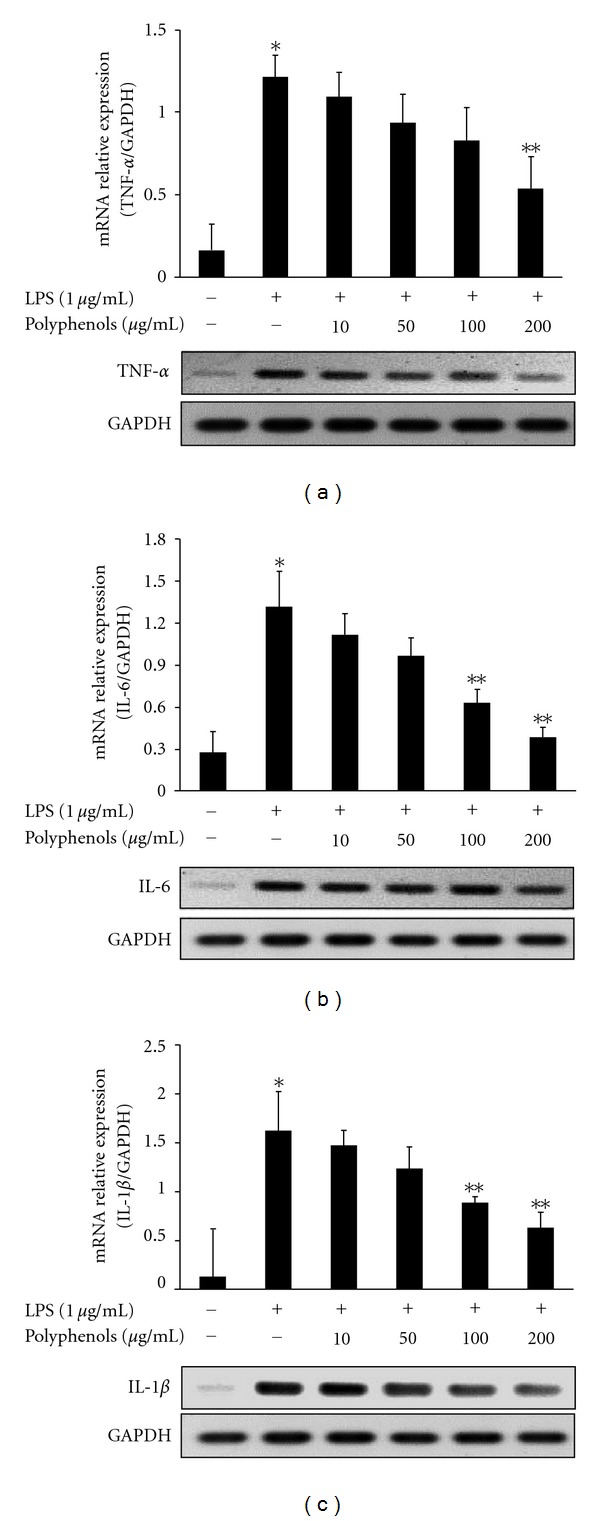
Effect of polyphenols on expression of pro-inflammatory cytokines. RAW 264.7 cells were treated with polyphenols extract at various concentrations (10, 50, 100, and 200 *μ*g/mL) for 1 h before treatment with LPS (1 *μ*g/mL) to induce inflammation. After 6 h of incubation, mRNA levels of (a) TNF-*α*, (b) IL-6, and (c) IL-1*β* were measured by RT-PCR. Data are the mean ± SD of triplicates. The asterisk (*) indicates a significant difference from the control group (*P* < 0.05) and **indicates significant difference from the LPS-treated group (*P* < 0.05).

To determine whether treatment with polyphenols affected the expression of pro-inflammatory cytokines, expressions of TNF-*α*, IL-1*β*, and IL-6 mRNAs were assessed by RT-PCR. When cells were treated with LPS (1 *μ*g/mL), all three mRNA levels significantly increased. However, mRNA expressions of TNF-*α*, IL-1*β*, and IL-6 were significantly decreased over that resulting from a single LPS stimulation by treatment of polyphenols at 200 *μ*g/mL and 100–200 *μ*g/mL, respectively (Figure 4).

### 3.6. Inhibition of Polyphenols on LPS-Induced Degradation and Phosphorylation of I*κ*B-*α* and Nuclear Translocation of the NF-*κ*B p65 Subunit in RAW 264.7 Cells

**Figure 5:**
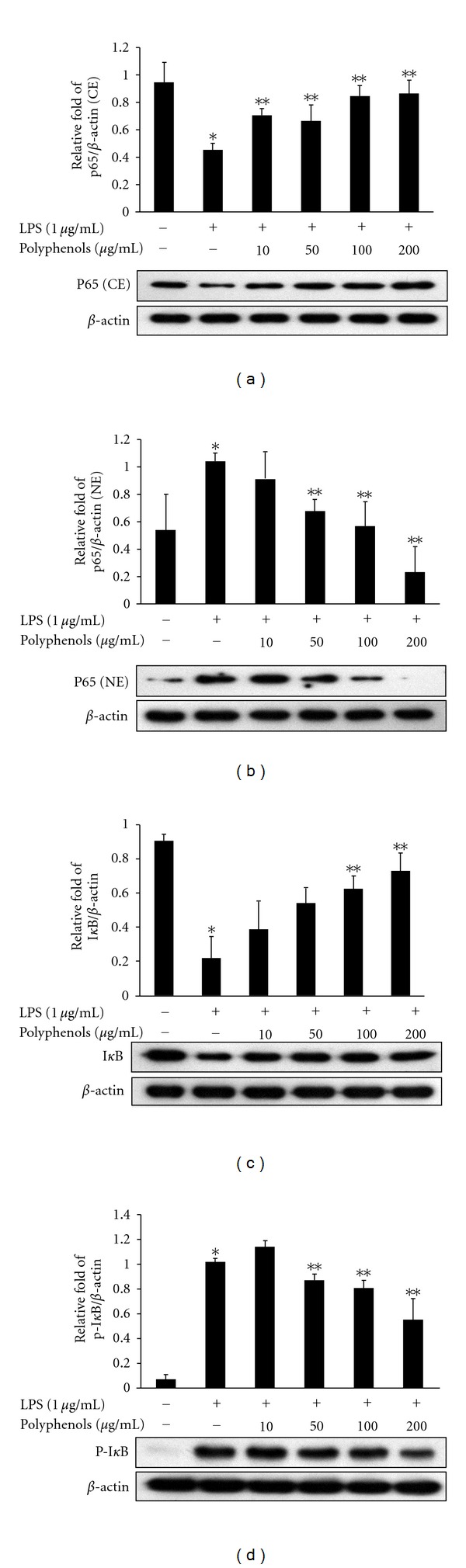
Effect of polyphenols on activation of NF-*κ*B in cytosol and nuclear translocation of NF-*κ*B p65. The cells were pretreated with the indicated concentrations of polyphenols for 1 h and treated with LPS (1 *μ*g/mL). After 30 min incubation, the expression of (a) cytosolic and (b) nucleic NF-*κ*B as well as (c) I*κ*B and (d) phosphorylated I*κ*B was examined by Western blot. Data are the mean ± SD of triplicates. The asterisk (*) indicates a significant difference from the control group (*P* < 0.05) and **indicates significant difference from the LPS-treated group (*P* < 0.05).

In order to understand the mechanisms underlying the inhibitory effect of polyphenols on LPS-induced inflammatory mediators such as COX-2, iNOS, and various cytokines, we further assessed the protein level of NF-*κ*B p65 in the cytosolic and nuclear fractions, as well as degradation and phosphorylation of I*κ*B-*α* by Western blot analysis. The amount of NF-*κ*B p65 in the nucleus was dramatically increased upon exposure to LPS alone and markedly decreased in the cytosolic fraction. However, in the nuclear fractions of RAW 264.7 cells, the expression levels of NF-*κ*B p65 protein were significantly attenuated by treatment with polyphenols at various concentrations (10, 50, 100, and 200 *μ*g/mL), suggesting that polyphenols inhibit the translocation of NF-*κ*B p65 protein from the cytosol to the nucleus (Figures 5(a) and 5(b)). Since the degradation and phosphorylation of I*κ*B-*α* are a principal step in NF-*κ*B activation by LPS, we examined the effect of polyphenols on LPS-induced degradation and phosphorylation of I*κ*B-*α* protein. I*κ*B-*α* was degraded in RAW 264.7 cells by a 30 min treatment with LPS, and this degradation was noticeably prevented by 100 and 200 *μ*g/mL of polyphenols (Figure 5(c)). The phosphorylated forms of I*κ*B-*α* were also barely detectable in the un-stimulated RAW 264.7 cells (Figure 5(d)). However, polyphenols inhibited LPS-induced phosphorylation of I*κ*B-*α* in a dose-dependent manner, similar to results from nuclear translocation of NF-*κ*B p65, while LPS alone led to a significant increase in the phosphorylation of I*κ*B-*α* (Figure 5).

### 3.7. Effect of Polyphenols on LPS-Induced Phosphorylation of MAPKs in RAW 264.7 Cells

**Figure 6:**
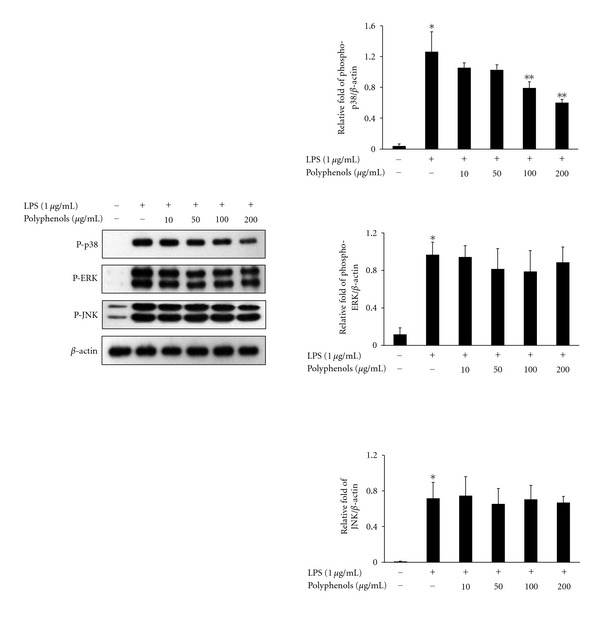
Effect of polyphenols on phosphorylation of MAPKs in RAW 264.7 macrophages. RAW 264.7 cells were pretreated with the indicated concentrations of polyphenols for 1 h and treated with LPS (1 *μ*g/mL) for 15 min. The cell lysates were analyzed by Western blot for the detections of phosphorylation of ERK1/2, JNK, and p38 MAPK. Data are the mean ± SD of triplicates. The asterisk (*) indicates a significant difference from the control group (*P* < 0.05) and **indicates significant difference from the LPS-treated group (*P* < 0.05).

Inflammation is triggered by intracellular signaling pathway events, which is related to the phosphorylation of MAPKs. To further understand the underlying possible mechanisms involved in the anti-inflammatory effect of polyphenols, we investigated whether polyphenols inhibited LPS-induced phosphorylation of MAPKs including p38 MAPK, ERK 1/2, and JNK. As shown in Figure 6, LPS treatment caused a strong increase in the phosphorylation of p38 MAPK, ERK 1/2. and JNK. However, co-treatment with 100 and 200 *μ*g/mL polyphenols reduced the LPS-induced phosphorylation of p38 MAPK, whereas the phosphorylation of ERK and JNK was not affected.

## 4. Discussion

Traditional Chinese medicine has used various herbal plants for centuries. A variety of compounds originating from herbal plants have various effects such as anticancer, anti-inflammation, and antiviral activities. Especially, *L. japonica T.* has long been used as a medicinal herb in Korea because of its anti-inflammatory and analgesic properties [15, 16]. *L. japonica T*. contains a hundred bioactive compounds including polyphenols. We identified 18 polyphenols isolated from Korea *L. Japonica T.* for investigating bioactive compound by using HPLC analysis. Among them, the dicaffeoylquinic acid is a major component and has anti-inflammatory effect by reducing the production of inflammatory mediators in lymphoma cells and suppressing COX-2 expression in macrophages [17].

Inflammation is a host primary response to infection or injury; macrophages and mast cells induce the release of inflammatory mediators such as COX-2, iNOS, and inflammatory cytokines. Normally, inflammation protects the host from external challenge, but prolonged inflammation causes various diseases. Inflammatory stimuli affect the four major intracellular signaling pathways: NF-*κ*B and the three MAPK pathways [18].

iNOS and COX-2 are important in the regulation of inflammation. COX is the target protein for analgesic and anti-inflammatory therapies that have been used for hundreds of years. The COX enzyme consists of at least two isoforms: COX-1 and COX-2. In mammals, the expression of COX-1 is constitutive in normal tissues such as production of prostaglandin precursors for thromboxane in platelets and the regulation of blood flow. In contrast to COX-1, COX-2 protein is only slightly expressed in most resting tissues. However, upon inflammatory stimulation, the expression levels of COX-2 protein and mRNA are increased [19]. Blocking NF-*κ*B and MAPKs activation by luteolin isolated from the flowers of *L. japonica* inhibits inflammatory mediators such as cytokines and COX-2 release from mast cells [20]. This study investigated that polyphenols suppressed the expressions of COX-2 protein and mRNA in LPS-induced RAW 264.7 cells (Figure 2). The results showed that the protein and mRNA expressions of COX-2 were decreased by polyphenols in LPS-induced RAW264.7 cells.

NOSs are a family of enzymes that catalyze the production of NO from L-arginine. Three isoforms of NOS have been found: neuronal NOS (nNOS), endothelial NOS (eNOS), and inducible NOS (iNOS). NOS exists in neuronal tissues (nNOS) and vascular endothelial cells (eNOS), whereas inducible NOS (iNOS) is detected in a variety of cell types including macrophages, microglial cells, astrocytes and keratinocytes. With infectious and proinflammatory stimuli, overexpression of iNOS protein and mRNA occurs. Poncirin, a flavanone glycoside isolated from *Poncirus trifoliata*, suppresses the expressions of iNOS, COX-2, and cytokines by blocking the NF-*κ*B pathway in LPS-induced RAW 264.7 macrophages [21]. Also, inactivation of NF-*κ*B by kaempferol downregulates iNOS and TNF-*α* expression in aged rat gingival tissues [22]. Our results showed that the levels of iNOS protein and mRNA were dose-dependently manner-suppressed by polyphenols in LPS-induced RAW 264.7 cells (Figure 3). Additionally, to investigate the anti-inflammatory effect by polyphenols, the mRNA levels of proinflammatory cytokines including TNF-*α*, IL-1*β*, and IL-6 were analyzed. This data represented that LPS-stimulated proinflammatory cytokine production was significantly attenuated by polyphenols in a dose-dependent manner (Figure 4). These results indicate that polyphenols suppressed the expression of iNOS and proinflammatory cytokines which were important role in inflammatory response.

NF-*κ*B plays a central role as an inducible transcription factor and is critical for regulation of gene expression in response to inflammation and immunity. NF-*κ*B proteins exist normally in the cytoplasm of inactivated cells, but these must be translocated into the nucleus to function in stimulated cells. The NF-*κ*B pathway is induced by LPS and pro-inflammatory cytokines such as TNF-*α* and IL-1*β* [23]. This process leads to the activation of RelA (p65)/p50 complexes. These complexes are controlled by the inhibitor of *κ*B (I*κ*B) proteins, which bind NF-*κ*B, thereby blocking nuclear translocation. When cells are exposed to extracellular stimuli, I*κ*B proteins are phosphorylated by IKK and separated from the NF-*κ*B complexes. The expressions of COX-2 and iNOS and synthesis of cytokines such as TNF-*α*, IL-1*β*, and IL-6 are controlled by NF-*κ*B [24]. In this study, determination of NF-*κ*B (p65) and I*κ*B protein expressions by Western blots showed that polyphenols increased the expression of p65 protein in the cytosol, whereas levels were decreased in the nucleus. Polyphenols inhibited the phosphorylation and degradation of I*κ*B (Figure 5). These results suggest that polyphenols inhibit NF-*κ*B nuclear translocation and activation by blocking I*κ*B degradation and phosphorylation in LPS-induced RAW264.7 cells. The data above represent that NF-*κ*B is a pivotal transcription factor for inflammatory signal pathway. Interestingly, our results demonstrate that polyphenols inhibit the expression of iNOS, COX-2, and cytokines via inactivation of NF-*κ*B by blocking I*κ*B degradation and phosphorylation.

MAPKs are comprised of three subtypes, including ERK, JNK, and p38 MAPK [25]. MAPKs mediate important signaling responses in the immune system and in the regulation of cellular activities including mitosis, cell proliferation and survival, and gene expression. The JNK and p38 MAPK pathways are activated by pro-inflammatory cytokines (IL-1 and TNF-*α*), microbial products (e.g., LPS), or cell stress; however, the extracellular signal-ERK pathway is activated by mitogenic stimuli. Activation of the p38 MAPK pathway is crucial for many immune response-related functions in macrophages [26]. The p38 MAPK pathway is involved in transcriptional regulation, but the exact mechanisms are uncertain. In previous studies, the pyridinyl imidazole SB203580, an inhibitor of p38 MAPK, inhibited the production of IL-6 and IL-8 mRNA or the induction of IL-6 and IL-8 in TNF-*α*- or IL-1*β*-induced rheumatoid synovial fibroblasts [27, 28]. The expressions of TNF-*α* and IL-1 are strongly regulated by p38 MAPK [29, 30]. MAPKs are involved in iNOS and TNF-*α* accumulation in stimuli-induced murine macrophages [31]. p38 MAPK and NF-*κ*B participate collaboratively in IL-6 gene expression and release in myocardial cells [32]. Also, the phosphorylation of p38 MAPK is regulated by LPS-induced NF-*κ*B activation [33]. To explore the regulation of MAPK by polyphenols, we investigated the phosphorylation of ERK, JNK, and p38 MAPK in LPS-stimulated RAW 264.7 macrophages. Polyphenols inhibited the expression of p38 protein, but not JNK and ERK, in LPS-induced RAW 264.7 cells (Figure 6). Therefore, these results suggest that polyphenols diminished the proinflammatory cytokines production, COX-2 and iNOS protein expression via the inhibition of p38 MAPK activation.

## 5. Conclusions

Our results demonstrate that polyphenol components isolated from Korea *L. japonica T. *have an anti-inflammatory activity through the inhibition of COX-2, iNOS, and cytokines such as TNF-*α*, IL-1*β*, and IL-6 by suppressing the p38 MAPK and NF-*κ*B pathways. This study may be the pharmacological basis of the use of polyphenols isolated from Korea *L. japonica T. *as a chemotherapeutic agent for the treatment of inflammatory diseases.

## Figures and Tables

**Table 1:** Concentration of polyphenols of *L. japonica THUNB.* (mg/kg)^a^.

Number	Quantity (mg/kg)	SD
1	898.28	1.54
2	131.46	0.52
3 + 4	721.88	0.70
5	18.03	0.14
6	521.66	8.36
7	5197.40	12.83
8	128.84	1.02
9	37.29	0.17
10	4451.10	10.66
11	16.16	0.14
12 + 13	37.58	0.14
14	61.10	1.03
15 + 16	71.33	0.82
17	154.25	1.63
18	123.00	1.27

^a^Data are the mean ± SD of triplicate determinations by HPLC-UV method at the 280 nm.
